# Predictive values of spinal cord diffusion magnetic resonance imaging to characterize outcomes after contusion injury

**DOI:** 10.1002/acn3.51855

**Published:** 2023-07-27

**Authors:** Rakib Uddin Ahmed, Daniel Medina‐Aguinaga, Shawns Adams, Chase A. Knibbe, Monique Morgan, Destiny Gibson, Joo‐won Kim, Mayur Sharma, Manpreet Chopra, Steven Davison, Leslie C. Sherwood, M.J. Negahdar, Robert Bert, Beatrice Ugiliweneza, Charles Hubscher, Matthew D. Budde, Junqian Xu, Maxwell Boakye

**Affiliations:** ^1^ Department of Neurological Surgery and Kentucky Spinal Cord Injury Research Center University of Louisville Louisville Kentucky USA; ^2^ Department of Anatomical Sciences and Neurobiology University of Louisville Louisville Kentucky USA; ^3^ Department of Neurosurgery Duke University Raleigh North Carolina USA; ^4^ Department of Radiology Baylor College of Medicine Houston Texas USA; ^5^ Department of Psychiatry Baylor College of Medicine Houston Texas USA; ^6^ Comparative Medicine Research Unit University of Louisville Louisville Kentucky USA; ^7^ Department of Radiology University of Louisville Louisville Kentucky USA; ^8^ Department of Neurosurgery Medical College of Wisconsin Milwaukee Wisconsin USA; ^9^ Clement J. Zablocki Veterans Affairs Medical Center Milwaukee Wisconsin USA

## Abstract

**Objectives:**

To explore filtered diffusion‐weighted imaging (fDWI), in comparison with conventional magnetic resonance imaging (MRI) and diffusion tensor imaging (DTI), as a predictor for long‐term locomotor and urodynamic (UD) outcomes in Yucatan minipig model of spinal cord injury (SCI). Additionally, electrical conductivity of neural tissue using D‐waves above and below the injury was measured to assess correlations between fDWI and D‐waves data.

**Methods:**

Eleven minipigs with contusion SCI at T8‐T10 level underwent MRI at 3T 4 h. post‐SCI. Parameters extracted from region of interest analysis included D_axial_ from fDWI at injury site, fractional anisotropy and radial diffusivity from DTI above the injury site along with measures of edema length and cord width at injury site from T_2_‐weighted images. Locomotor recovery was assessed pre‐ and weekly post‐SCI through porcine thoracic injury behavior scale (PTIBS) and UD were performed pre‐ and at 12 weeks of SCI. D‐waves latency and amplitude differences were recorded before and immediately after SCI.

**Results:**

Two groups of pigs were found based on the PTIBS at week 12 (*p* < 0.0001) post‐SCI and were labeled “poor” and “good” recovery. D‐waves amplitude decreased below injury and increased above injury. UD outcomes pre/post SCI changed significantly. Conventional MRI metrics from T_2_‐weighted images were significantly correlated with diffusion MRI metrics. D_axial_ at injury epicenter was diminished by over 50% shortly after SCI, and it differentiated between good and poor locomotor recovery and UD outcomes.

**Interpretation:**

Similar to small animal studies, fDWI from acute imaging after SCI is a promising predictor for functional outcomes in large animals.

## Introduction

Spinal Cord Injury (SCI) currently affects 17,000 people in the US annually.^1^ Despite attempts to prognosticate SCI, conventional magnetic resonance imaging (MRI) remains limited. Accurate clinical assessment is essential for optimizing initial therapies to predict the outcomes. However, this is unreliable in some patients. Many advanced neuroimaging studies have been performed in rodents. However, significant anatomical, functional, molecular, and pathological differences between rodent and human spinal cords have also been cited as possible reasons for the lack of translation to clinical practice.^2^, ^3^, ^4^ Moreover, an interest in the large animal model is more closely aligned because of the similarity in size compared to the human spinal cord.^4^, ^5^ Advantages of this model include large cord size, cerebrospinal fluid/cord ratios, neuroanatomical similarity to the human spinal cord, and physiological similarities to humans.^6^, ^7^, ^8^, ^9^, ^10^, ^11^ The sizeable spinal canal and cord allow for the evaluation of advanced spinal cord imaging methods applicable to humans.

MRI is valuable in the evaluation of SCI. The presence of hemorrhage and length of edema are predictive of SCI outcomes.^12^, ^13^, ^14^, ^15^, ^16^ Despite its obvious utility in acute SCI evaluation, conventional MRI is limited in many ways. These limitations include the inability to provide information about axonal injury and electrical conductivity. Axonal injury is considered a more accurate predictor of neurological outcome.^17^ Diffusion tensor imaging (DTI) is an MRI technique that has shown promise in estimating the degree of axonal injury in acutely injured cords in animal^18^, ^19^, ^20^, ^21^ and some human studies.^17^, ^22^, ^23^ In addition, fractional anisotropy (FA), a key DTI parameter that is reduced in acute SCI, is confounded by edema and hemorrhage, making its interpretation and reliability in critical SCI evaluation problematic. To circumvent these issues at the site of injury, DTI monitored remotely from the site of injury has been demonstrated to be related to the severity of SCI in patients.^24^, ^25^ Separately, filtered diffusion‐weighted imaging (fDWI) is based on the principles of double diffusion encoding (DDE), which minimizes signals from extracellular water to more directly probe intra‐axonal diffusion parallel to axons as a marker of early acute beading. fDWI in rodent studies showed good prognostication of functional recovery.^26^, ^27^ However, it has only been reported as a proof‐of‐principle in a human study^28^ without assessment of outcomes and has not been demonstrated in a large animal model. In particular, there remains a need for MRI imaging biomarkers of urinary function, since this domain is one of the primary areas most important to patients with SCI.^29^ Many efforts have aimed to classify and characterize neurogenic lower urinary tract dysfunction (NLUTD) after SCI.^30^, ^31^, ^32^, ^33^, ^34^ Recently, attempts to use neuroimaging diagnostic tools aimed to predict long‐term urinary dysfunction have been made. The current data, however, are very limited, and more studies are needed.^33^

Following electrical stimulation aimed at the motor cortex, D‐waves reflect the connectivity from motor cortex to the spinal cord via corticospinal tract. D‐waves measured from the epidural space have received little attention as a potential electrophysiological marker of injury severity. D‐waves represent descending motor‐evoked potentials from activated fast‐conducting corticospinal tract following a transcranial electrical stimulus targeting motor cortex.^34^, ^35^, ^36^, ^37^ In studies comparing epidural D‐waves to muscle motor evoked potentials, D‐waves are more predictive of clinical outcome.^34^, ^35^, ^38^ In cases when muscle motor evoked potentials were absent intraoperatively, the presence of a D‐wave below the lesion accurately predicted favorable prognosis.^39^ However, there is limited data on the value of D‐wave monitoring in acute SCI and its correlation with MRI imaging.

The overall goal of the present pilot study was to use the Yucatan minipig model to explore fDWI, in comparison with conventional MRI and DTI, as a clinical predictor for long‐term locomotor and urodynamic outcomes. Additionally, the electrical conductivity of neural tissue using D‐waves above and below the injury was measured to assess corrections between fDWI and electrophysiological data.

## Materials and Methods

### Animals

This study was approved by the University of Louisville (UofL) Institutional Animal Care and Use Committee (IACUC) and conducted in accordance with the Guide for the Care and Use of Laboratory Animals, 8th edition and the Animal Welfare Act and Regulations. The UofL animal care program is accredited by the Association for the Assessment and Accreditation of Laboratory Animal Care, International (AAALAC). Eleven 5‐month‐old female Yucatan miniature swine (Sinclair BioResources, Auxvasse, MO, USA) with weights of 17–21 kg were used for this study.

### Locomotor training

After acclimation, animals began positive reinforcement training with a clicker.^40^ Training consisted of three to four 10–20‐min sessions for a maximum of one hour per day for three weeks. The animals were trained to task with the end goal of walking straight over a rubber mat.

### Sling training

Once the animals finished clicker and target training, sling training was started to assess urinary tract function with urodynamics.^41^ Once the animal was acclimated to the presence and sound of the sling, it was picked up and placed within it. The trainer sat in front of the animal and provided positive reinforcement in the form of food treats. Other assistants ensured the animal was secured in this sling and offered positive reinforcement. The animal remained in the sling for 15 min during the first session, with the time increased by 5 min every session. After approximately 4 sessions, the animal could sit calmly in the sling for 45 min.

### Anesthesia and surgical procedures

To induce the anesthesia, Ketamine (5 mg/kg IM), Dexmedetomidine (0.04 mg/kg IM), and Glycopyrrolate (0.01 mg/kg IM) were mixed and administered as described before.^42^ Subsequently Bupivacaine SR (2 mg/kg SQ) was administered into several locations near the incision site. Meloxicam (0.4 mg/kg IM) was administered for 3 days. Following endotracheally intubation, anesthesia was maintained with Propofol (2–20 mg/kg/hour IV) and Fentanyl (10–45 mcg/kg/hour). To maintain the continence an indwelling urinary catheter (8 French foley) was inserted until the animal urinate independently. A heating pad was used to maintain the body temperature at 99.1–103 °F (Bair Hugger Model 775, 3M, Saint Paul, MN, USA).

In ventral recumbency condition a dorsal midline incision was made between T8 and T13 to expose the spinous processes, laminae and pedicles. Laminectomy was performed at T10 level to induce contusion compression injury. In the pedicle two multi‐axial screws were inserted at T11 and T13. Two titanium rods were used to articulate the arm of the impactor and screws and fixed the vertebral segments. To induce contusion SCI a 50‐gm weight impactor dropped from variable heights onto the spinal cord with a mass of 100 gm to cause the compression. Fentanyl patches and Maropitant citrate continued for 3 days postoperatively from day of injury. Propofol was discontinued and replaced with isoflurane (1%–2%) prior to and during MRI imaging on the day of surgery.

### Postoperative care

Following SCI, the animals were monitored continuously by critical care veterinary staff under the direct supervision of a veterinarian for approximately 7 days until the animals met criteria to return to regular housing, which included the ability to ambulate, intake food & water, maintain normal urine output, and maintain normal body temperature. The urinary catheters remained in place until animals voided urine around the urinary catheter (either reflexively or volitionally). Analgesia was continued with fentanyl transdermal patches for 6 days post‐operatively and meloxicam IV or PO once daily for 3 days post‐operatively. Blood glucose and potassium were monitored and supplemented IV as needed. Cephalosporin antibiotics were continued for 7 days post‐operatively or until urinary catheters were removed.

### Behavioral assessments

**Figure 1:**
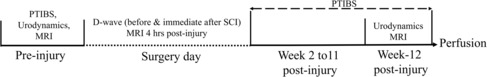
Experimental procedure. At pre‐injured condition, Yucatan minipigs were trained to perform the locomotor to record the porcine thoracic injury behavior scale (PTIBS) (*n* = 11) as well as sling training for urodynamic outcomes (*n* = 8). MRI was recorded from the pigs (*n* = 5) prior to the surgery day. On surgery day, before and immediately after 10 min of SCI, D‐waves (*n* = 8) were recorded. 4 h post‐injury the MRI were done in all pigs (*n* = 11). After recovering from surgery, PTIBS were recorded weekly from week 2 to week‐12 (*n* = 11). Finally, at week‐12, before the perfusion, urodynamics (*n* = 8) and MRI (*n* = 5) were recorded from the pigs.

Locomotion behavior outcome was collected using the PTIBS at 1 week before the injury and starting a week after injury until 12 weeks (*n* = 11) (Fig. 1). PTIBS is a 10‐point scale that describes stages of recovery of hindlimb function after SCI while attempting to walk in a straight line over a rubber mat.^8^ PTIBS >7‐point scale is considered a “good recovery” and <3‐point a “poor recovery”.

### MRI methods

MRI was performed on a Siemens Skyra 3T at the MRI research facility at the University of Louisville with custom pulse sequence developed in collaboration with the Baylor College of Medicine and the Medical College of Wisconsin. Eleven minipigs were scanned after 4 h post thoracic contusion injury (short‐term) (Fig. 1). Five animals were scanned again after 12 weeks post‐injury (long‐term) (Fig. 1). Three anatomic sequences were acquired: T_1_‐weighted, T_2_‐weighted, and short tau inversion recovery (STIR). Each sequence was performed with a large sagittal field of view (FOV) to evaluate the whole spine, a sagittal small FOV for higher resolution to cover the site of injury, and several axial locations at the injury site.

DTI was collected superior to the injury site centered at the T3 vertebra, including 12 axial slices with 5 mm thickness each using an inner‐volume echo planar imaging sequence^43^ with multiband (MB = 2) excitation at 0.78 × 0.8 mm in‐plane resolution (FOV = 86 × 24 mm, matrix size = 110 × 30), slice thickness = 5 mm, TE = 61.4 ms, echo spacing = 1.48 ms, bandwidth = 758 Hz/Px, phase partial Fourier = 6/8. DTI included two polarity reversed 25 unique diffusion directions with monopolar diffusion encoding and b‐values distributed between 300–800 s/mm^2^, in addition to four b0 images interleaved into the diffusion encoding table (a total of 54 volumes). Each slice in the DTI acquisition was gated to an oxygen saturation signal (SpO_2_) to reduce motion artifact with 200 ms delay and an acquisition window of 700 ms, resulting in TR ~ 5 s. The DTI acquisition was repeated 5 times with a total acquisition time of ~40 min. fDWI was collected without multiband excitation with 6 axial slices centered at the injury site using the same acquisition parameters, except for TE = 81 ms due to a simplified filter‐probe diffusion encoding scheme (one case, PIG2480, was centered at out‐of‐injury site). Diffusion directions for fDWI started with a vector perpendicular to the spinal cord (left–right) with b value of 1350 s/mm^2^, followed by four vectors tilting and increasing this initial vector towards parallel to the cord axis by incrementally adding diffusion weighing in the slice direction (b‐values = 1463, 1575, 1688 and 1800 s/mm^2^). Each fDWI acquisition consisted of (four possible combinations of) polarity reversed repetitions of this 5‐direction filter‐probe diffusion encoding, in addition to one b0 image (a total of 21 volumes). The fDWI acquisition was repeated 3–5 times with a total acquisition time of ~13–20 min. Five unique directions were used for fDWI with a simplified filter‐probe table, by slightly tilting a high b‐value vector perpendicular to the spinal cord towards the cord axis (i.e., incrementally adding small diffusion weighting in the slice direction). The concept of fDWI is to use the high b‐value diffusion gradients perpendicular to the spinal cord to suppress extracellular water signals which reduces the confounding effects of vasogenic edema prominent in SCI (in addition to eliminating CSF partial volume effects). The diffusion directions applied with lower b‐values parallel to the cord are used to quantify the parallel/axial diffusivity that reflects intra‐axonal hindrances. This concept is illustrated graphically in a previous human study of SCI.^44^ Further, that study demonstrated high‐quality fDWI at 3T (research scanner) in the normal human cervical cord and at 1.5T in several cases of acute cervical spinal cord injury (clinical scanner). In our experience, fDWI often results in fewer artifacts since the suppression of CSF and muscle reduces motion and pulsation artifacts (ghosting) that can hinder typical DWI/DTI. Since it uses product sequences for DWI but with custom encoding directions/b‐values, it is also compatible with reduced field of view excitation methods available through most vendors to reduce susceptibility artifacts. The lower SNR of fDWI is the major drawback, though, for which we had performed pilot imaging studies to ensure sufficient SNR in our current fDWI imaging protocol.

The DTI and fDWI image volumes were registered using 2D x‐ and y‐translations on each axial slice. Diff_4dfp generated the DTI maps of fractional anisotropy (FA), mean diffusivity (MD), axial diffusivity (AD), and radial diffusivity (RD) in 4dfp tools (https://4dfp.readthedocs.io/en/latest/index.html) and the fDWI D_axial_ and D_radial_ maps were generated by in‐house MATLAB scripts. For DTI analysis, anterior white matter (WM), left and right corticospinal tract (CST), posterior column (PC), and gray matter (GM) regions of interest (ROIs) were manually defined based on mean b0 images, mean diffusion‐weighted images, and DTI FA maps. Then, the left and right CST ROIs were merged, and the DTI measures averaged across slices after confirming no statistically significant difference in the DTI measures among the left and right CST and across slices. For fDWI analysis, whole spinal cord regions of interest (ROI) were manually defined based on mean diffusion‐weighted images, which show clear boundary of the spinal cords. We first applied thresholding on the mean images and then manually modified the ROIs. To avoid partial volume effects, we excluded inconclusive boundary voxels (conservative segmentation). The medians of fDWI measures at each slice were used as representative measures, and the lowest D_axial_ value (Min. D_axial_) across the slices was used for subsequent statistical analysis. Conventional MRI images were quantified in a manner consistent with the NIH CDE recommendations for spinal cord injury, which include: the extent and length of edema (mm), and cord width (mm).^45^ One subject was excluded from fDWI analysis because the slice location was out of the injury site.

### D‐waves

D‐waves were recorded before and 10 min after SCI.^46^ To stimulate the motor cortex, two 5 cm small skin incisions were placed in the scalp overlaying the skull. Two stimulation trains of stimulus intensities of 50–400 V (pulse duration 1.0 ms, ISI 0.5 ms) were delivered through stainless steel alligator clips clamped to the screws, which were placed according to the international 10–20 nomenclature: FC1, FC2 was assigned to points 7.5 mm towards the nasion from the vertex and 5 mm lateral to the midline. Two double contact strip electrodes (Cortac Electrodes, PMT Corporation) were placed in the exposed dura at dorsal epidural space 1 cm cranial and 1 cm caudal to the injury site. D waves were recorded by stimulating the motor cortex before and after the spinal cord contusion injury. All the signals recorded were amplified and filtered (50.0 Hz low frequency, 1 kHz high frequency, Nim Eclipse, Medtronic, MN, USA). Amplitude and latencies were recorded before and after the SCI from above and below the injury site. After recording the data, the holes were filled with bone wax and the incision was closed using absorbable continuous subcuticular suture.

### Urodynamic analysis

After successful sling training, the minipig's bladder function was evaluated at pre‐(*n* = 8) and 12‐weeks post‐injury (*n* = 8) under awake, non‐sedated conditions (Fig. 1). To evaluate bladder function, a sterile T‐Doc® air‐charged 7Fr single sensor with filling lumen (CAT895, Laborie, VT, USA) was manually inserted into the bladder through the urethra after aseptic preparation of the perineal area with chlorhexidine scrub. The urinary bladder was filled with saline at a rate of 15 mL/min.^47^ Another T‐Doc® air‐charged 7Fr abdominal catheter (CAT875, Laborie, VT, USA) was also placed in the rectum. To record the EMG activity, two surface electrodes (Neotrode® II, 1741‐003, Conmed, NY, USA) were placed near the perineal region. Functional bladder capacities (FBC), void duration (VD), voided volume (VV), post‐void residual (PVR), voiding efficiency (percentage of volume voided) compared to the pre‐void bladder volume $VE=\left(\frac{\mathrm{VV}}{\mathrm{VV}+\mathrm{PVR}}\right)\times 100$; average flow rate, $\mathrm{AFR}=\left(\frac{\mathrm{VV}}{\mathrm{VD}}\right)$ were measured at pre‐and week‐12 post‐injured condition.

### Statistical methods

PTIBS outcomes were summarized as mean and standard deviation (SD). D‐wave outcomes (latency and amplitude above and below injury) were evaluated by mixed linear models with a random intercept to account for individual variability. Independent variables in the models were recovery type (poor/good), timepoint (pre/post), and their interaction. Least square means and standard errors were used to summarize D‐waves outcomes. Different comparisons were performed through linear contrasts built on the interaction term. The urodynamic parameters (FBC, VD, VV, PVR, VE, and AFR) were evaluated using paired *t*‐test to compare the outcomes before and after 12 weeks of SCI. Conventional MRI analysis of good vs. poor recovery groups were performed using the Mann–Whitney test. The associations of conventional MRI, fDWI, and DTI to PTIBS, D‐waves, and urodynamics outcomes were evaluated with linear regression and summarized with the least square mean estimates with associated standard errors. Models were evaluated with R‐squared. In cases where 2 or more imaging modalities predicted the same outcome, the 2 models were compared using the F‐test. Correlation analysis was performed between conventional MRI, fDWI, and DTI using Pearson correlation coefficient. Statistical analyses were performed in SAS 9.4 (SAS Inc, Cary, NC), and Prism 9 (GraphPad, San Diego, CA).

## Results

### PTIBS

**Figure 2:**
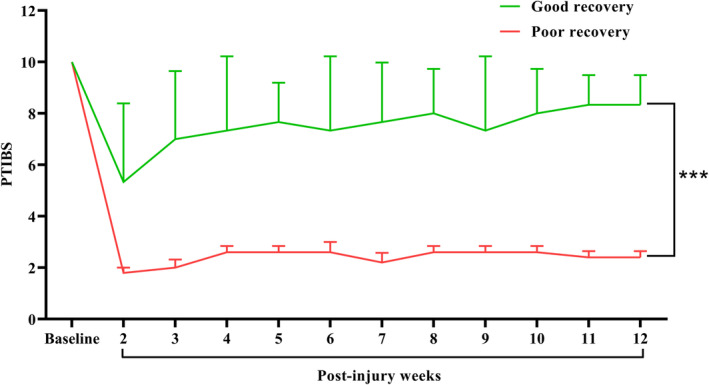
PTIBS scores. Significant differences were observed in between the good (*n* = 5) and poor recovery (*n* = 6) animal (***p < 0.001). Data presented as Mean ± SD.

After two weeks of recovering from the injury, PTIBS were recorded weekly. Based on this locomotor recovery, pigs were classified into two recovery groups: poor locomotor (<3 point) and good locomotor recovery (>7 point). At 12 weeks post‐injury significant differences were observed between two group of pigs (3 ± 1 vs 7 ± 1; *p* < 0.001) (Fig. 2).

### Force impact

Based on PTIBS, injury information's are summarized in Table 1. Significant differences were observed in good and poor recovery pigs force (1037 ± 371.7 vs 2616 ± 500.2; *p* = 0.0015), displacement (0.58 ± 0.3184 vs 1.633 ± 0.4402; *p* = 0.0486), impulse (4.796 ± 1.387 vs 8.580 ± 1.332; *p* = 0.0030) and velocity (626.4 ± 314.7 vs 1389 ± 265.7; *p* = 0.0001).

**Table 1:** Summary of biomechanical parameters of injury.

Pig ID	Drop height	Level	Weight (kg)	Force (kdynes)	Displacement (mm)	Impulse (kdynes × sec)	Velocity (mm/sec)	Compression time
**Good recovery**								
3226	5 cm	T10‐T11	22.8	746.5	0.6	4.51	667	5 min
3640	3.5 cm	T9‐T10	26	1012.4	0.3	4.47	331	‐
3923	2 cm	T9‐T10	20.1	428.7	0.1	2.32	149	2 min
3924	2 cm	T9‐T10	23	529.3	0.1	2.64	157	2 min
2958	20 cm	T9‐T10	27.3	2469.17	1.8	10.04	1828	5 min
Mean ± SEM			23.84 ± 1.273	1037 ± 371.7	0.58 ± 0.3184	4.796 ± 1.387	626.4 ± 314.7	
**Poor recovery**								
2480	20 cm	T8‐T9	26	4042.27	3.4	11.5	1826	5 min
2933	20 cm	T9‐T10	26.35	3479.13	1.7	10.69	1756	5 min
2938	20 cm	T10‐T11	25.5	2987.98	1.9	9.99	1770	5 min
3228	20 cm	T9‐T10	20.3	2949.26	1.8	10.2	1868	5 min
2468	5 cm	T9‐T10	26	1094.6	0.4	5.8	429	5 min
2959	5 cm	T9‐T10	24	1145.02	0.6	3.3	685	5 min
Mean ± SEM			24.69 ± 0.9415	2616 ± 500.2*	1.633 ± 0.4402*	8.580 ± 1.332*	1389 ± 265.7*	

Data presented as Mean ± SEM.

* *p* < 0.05.

### fDWI at injury site

**Figure 3:**
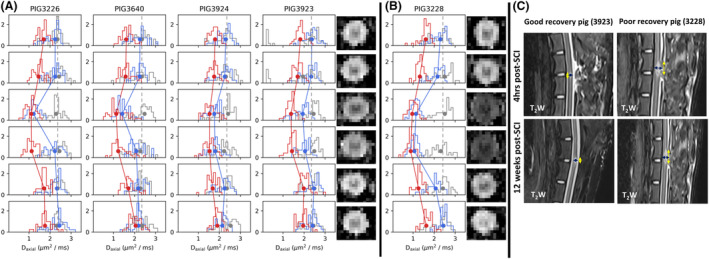
fDWI D_axial_ distributions of good recovery cases (A) and a poor recovery case (B) and representative D_axial_ maps. The rows are slices. The histograms are the distributions of D_axial_ measures in the corresponding slice's whole spinal cord ROI. The y axes are probability density (i.e., the area under each histogram is 1). The gray, blue, and red colors represent preinjury, short‐term, and long‐term. The dots are median values in the ROI and they were connected by line segments for visualization. (C) Conventional MRI lesion of two representative pigs from two different groups at 4 h post‐SCI and 12 weeks post‐SCI. The yellow line indicates the edema length and the dark blue line represents the cord width.

Pre‐injury median D_axial_ and D_radial_ values (*n* = 5) ranged from 1.95 to 2.54 and from 0.28 to 0.40 μm^2^/ms, respectively. Those at short‐term (*n* = 10) ranged from 0.81 to 2.49 and from 0.26 to 0.53 μm^2^/ms, while those at long‐term (*n* = 5) ranged from 0.88 to 2.05 and from 0.52 to 1.34 μm^2^/ms, respectively. For the 5 animals with longitudinal studies, D_axial_ was diminished on the injury site after injury (short‐term) and on all 6 acquired slices at long‐term (Fig. 3), while D_radial_ did not appreciably change at short‐term but changed more at long‐term (Fig. S1).

### DTI rostral to the injury site

The means ± SD of FA, AD, RD, and MD on each ROI were summarized in Table 2. The longitudinal changes in DTI measures of the good and poor recovery groups as well as representative DTI maps and ROIs were presented in Fig. S2.

**Table 2:** The means ± SD of DTI metrics FA, AD, RD, and MD on anterior WM, CST, PC, and GM at preinjury (*n* = 5), short‐term (*n* = 20), and long‐term (*n* = 9).

Timepoint	Region	FA	AD (μm^2^/ms)	RD (μm^2^/ms)	MD (μm^2^/ms)
Pre‐injury (*n* = 5)	Anterior WM	0.70 ± 0.03	1.96 ± 0.11	0.50 ± 0.05	0.99 ± 0.06
	CST	0.77 ± 0.05	2.08 ± 0.29	0.39 ± 0.02	0.96 ± 0.09
	PC	0.73 ± 0.03	1.93 ± 0.21	0.45 ± 0.04	0.94 ± 0.08
	GM	0.59 ± 0.03	1.69 ± 0.14	0.57 ± 0.01	0.95 ± 0.05
4 h post‐injury (*n* = 11)	Anterior WM	0.71 ± 0.04	2.03 ± 0.12	0.50 ± 0.08	1.01 ± 0.08
	CST	0.79 ± 0.04	1.95 ± 0.19	0.36 ± 0.05	0.89 ± 0.08
	PC	0.72 ± 0.04	1.89 ± 0.18	0.46 ± 0.08	0.94 ± 0.10
	GM	0.61 ± 0.03	1.72 ± 0.11	0.57 ± 0.06	0.96 ± 0.07
12 weeks post‐injury (*n* = 5)	Anterior WM	0.71 ± 0.04	2.02 ± 0.08	0.50 ± 0.05	1.01 ± 0.03
	CST	0.75 ± 0.01	1.91 ± 0.15	0.42 ± 0.04	0.92 ± 0.08
	PC	0.72 ± 0.02	1.93 ± 0.14	0.47 ± 0.04	0.96 ± 0.07
	GM	0.62 ± 0.04	1.79 ± 0.07	0.57 ± 0.06	0.98 ± 0.04

### Conventional MRI analysis

**Figure 4:**
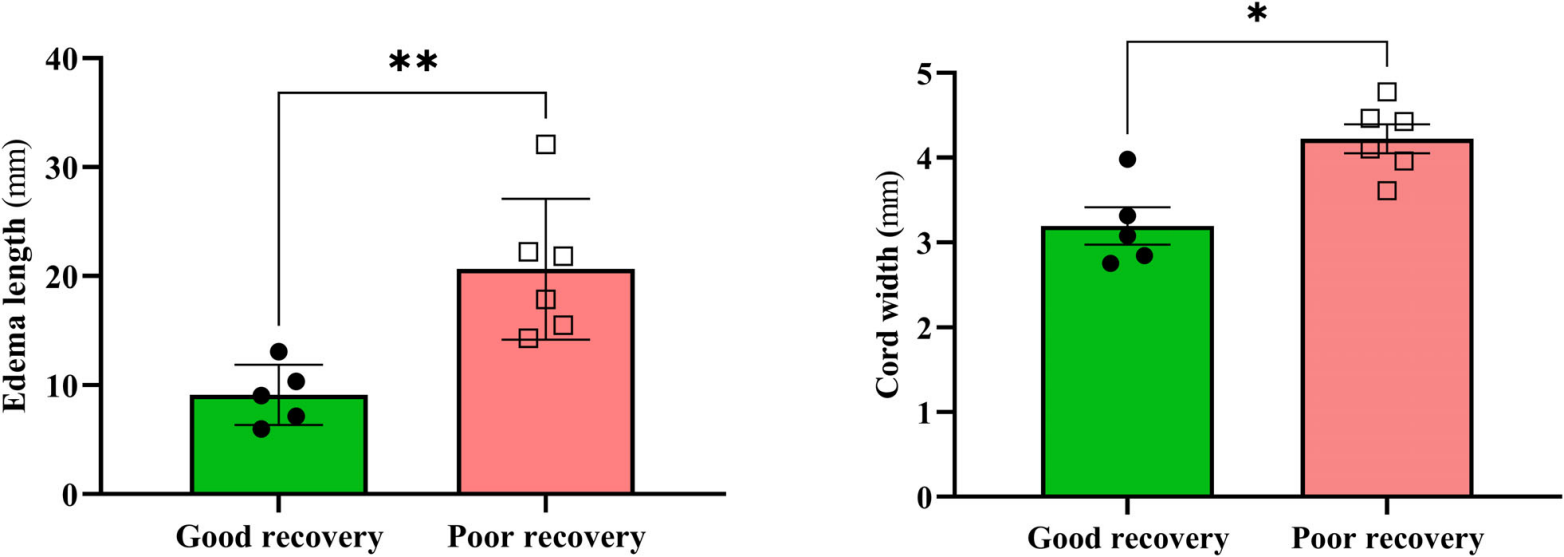
MRI lesion differences between good (*n* = 5) and poor recovery (*n* = 6) pigs at 4 h post‐injury. Data presented as Mean ± SD; *p < 0.05, **p < 0.01.

At 4‐h post‐injury the edema length and cord width were measured to see the differences between good and poor recovery pigs. Significant differences were found in good and poor recovery pigs edema length (9.127 ± 2.773 vs 20.64 ± 6.458; *p* = 0.0043) and cord width (3.193 ± 0.4931 vs 4.224 ± 0.4163; *p* = 0.0173) (Fig. 4).

### D‐waves outcomes

**Figure 5:**
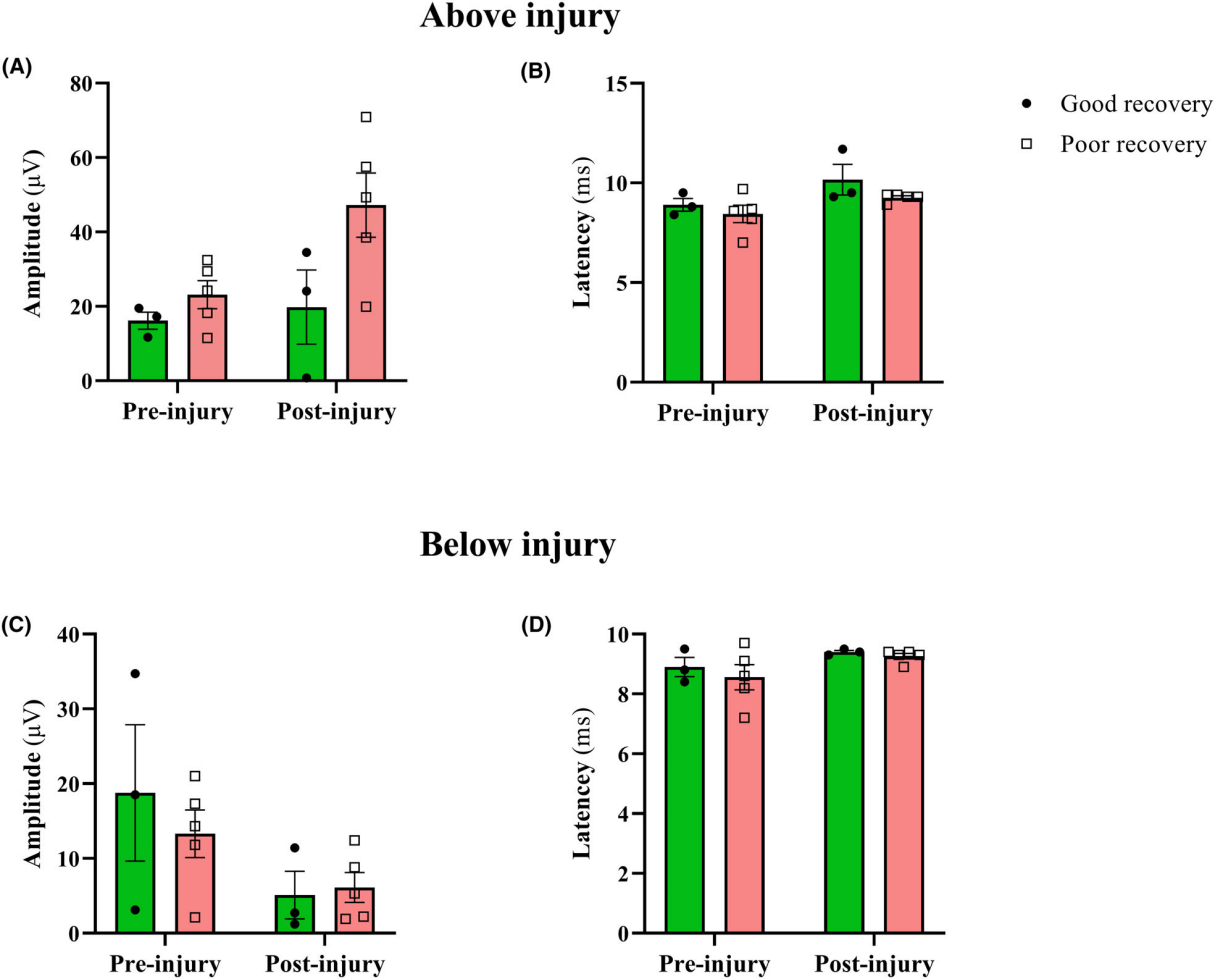
D‐waves outcomes at pre‐ and immediate after injury at above and below the injury level. (A) In good recovery pigs the amplitude increased immediate after injury (19.79 ± 9.98) compared to pre‐injury (16.13 ± 2.31) condition (*p* = 0.0824). In poor recovery pigs the amplitude was found higher after injury (47.22 ± 8.64) compared to pre‐injury (23.14 ± 3.78, *p* = 0.1880). (B) No significant changes of latency were found in good (8.90 ± 0.32 vs 10.17 ± 0.77, *p* = 0.6093) and poor recovery (8.44 ± 0.44 vs 9.26 ± 0.09, *p* = 0.1343) pigs. (C) Below injury level amplitude dropped in good recovery pigs (18.77 ± 9.12 vs 5.10 ± 3.18, *p* = 0.4685). However, in poor recovery pigs, it increased a little (5.1 ± 3.19 vs 6.2 ± 2.06, *p* = 0.6171). (D) No significant changes were observed in good (8.90 ± 0.32 vs 9.40 ± 0.05, *p* = 0.7572) and poor recovery (8.56 ± 0.42 vs 9.26 ± 0.09, *p* = 0.4568) pigs. Data presented as Mean ± SEM.

D‐waves were recorded in good and poor recovery pigs above and below the injury level. Amplitude changes (Fig. 5A,C) were found in both groups of pigs at above and below the injury level. However, there was no significant difference in the latency (Fig. 5B,D). The findings suggested that corticospinal tract remains functional, and the injury is incomplete at the level of T8‐T10.

### Urological outcomes

**Figure 6:**
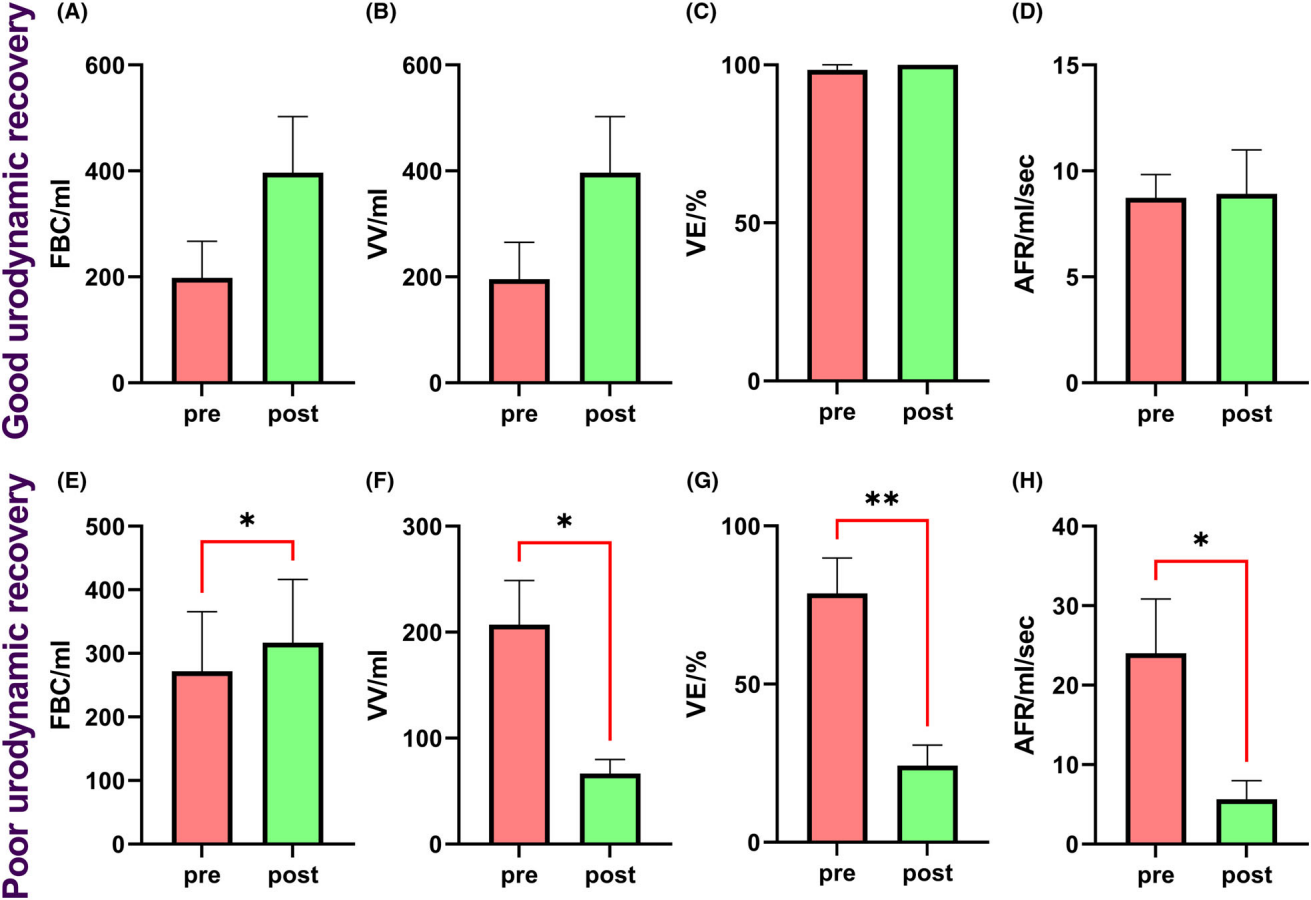
Statistical analysis of the changes of the urodynamic parameters after 12 weeks post‐SCI. After 12 weeks post‐injury one group of animals (*n* = 3) was classified as good recovery based on the urodynamic outcomes at 12 weeks post injury (A–D). The second group (*n* = 5) was classified as poor recovery based on the same criteria (E–H). The good recovery animals showed non‐significant increases on the functional bladder capacity (FBC, A) and voided volume (VV, B), while any change was found in terms of voiding efficiency (VE, C) or average flow rate (AFR, D). In the poor recovery animals, it was observed a significant increase on FBC (E, *p* = 0.04) and a decrease on the VV (F, *p* = 0.03), VE (G, *p* = 0.003), and AFR (H, *p* = 0.01). Bars represent the mean ± SEM and the brackets highlight statistical significance resulting of a paired *t*‐test.

The animals were divided in two separated groups based on the motor recovery (poor locomotor recovery and good locomotor recovery) and the urodynamic parameters were compared between groups. The good locomotor recovery animals show a non‐significant increase on the VV (Fig. 6B, *p* = 0.2515) and FBC (mL) (Fig. 6A, *p* = 0.2910), while no changes were found on the VE (%) (Fig. 6C *p* = 0.5566) and AFR (mL/s) (Fig. 6D, *p* = 0.0799). The poor locomotor recovery animals show a significant increase on the FBC (mL) (Fig. 6F, *p* = 0.04), while significant reduction on the VV (mL) (Fig. 6F, *p* = 0.03), VE (%) (Fig. 6G, *p* = 0.003) and AFR (mL/s) (Fig. 6H, *p* = 0.01) were observed.

### Correlation of conventional MRI with fDWI and DTI

**Figure 7:**
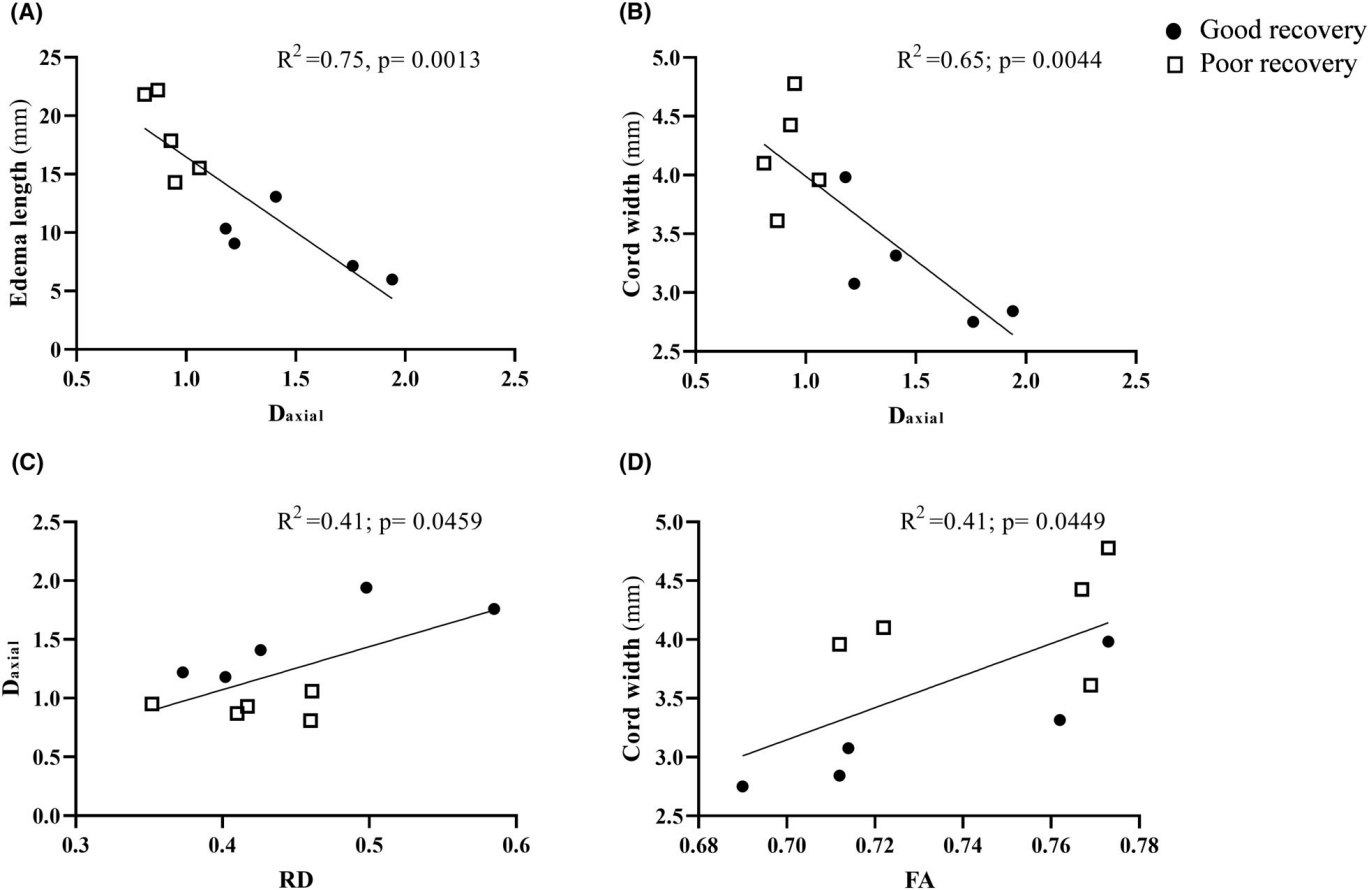
Correlation of conventional MRI, fDWI, and DTI. Conventional MRI significantly correlated with fDWI derived D_axial_ (A and B), DTI metrics FA (D) and fDWI value correlate with the DTI metrics RD (C) 4 h post‐injury.

Correlation analyses revealed a strong negative correlation between fDWI‐derived D_axial_ at the injury epi‐center and conventional MRI edema length (*R* ^2^ = 0.75; *p* = 0.0013) (Fig. 7A) and cord width (*R* ^2^ = 0.65, *p* = 0.0044) (Fig. 7B). fDWI derived D_axial_ correlated with the DTI metrics RD (*R* ^2^ = 0.41, *p* = 0.0459) (Fig. 7C). DTI metrics FA in the spinal cord white matter rostral the injury site showed significant correlation with cord width (*R* ^2^ = 0.41, *p* = 0.0449) (Fig. 7D).

### Predictive association of fDWI, DTI, and conventional MRI with PTIBS, D‐waves, and urodynamics

On the 4‐h post‐injury conventional MRI, a 1 mm increase in edema length was associated with a 0.32 decrease in post‐injury PTIBS (SEM: 0.09, *p*: 0.0059, *R* ^2^: 0.59) and 1 unit increase in cord width of the same MRI was associated with a −3.95 change in post‐injury PTIBS (SEM: 0.87, *p*: 0.0014, *R* ^2^: 0.70). fDWI was also a predictor of PTIBS with 1 unit of D_axial_ being associated with a preservation of 6.78 in post‐injury PTIBS (SEM: 1.79, *p*: 0.0053, *R* ^2^: 0.64). The best predictor of PTIBS is conventional MRI cord width, explaining about 70% of variance, followed by D_axial_ explaining 64% of variance. However, further analysis showed that the 2 predictors were statistically comparable (F‐test comparing the edema length and D_axial_: 0.78, numerator DF: 8, denominator DF: 9, *p*: 0.63; and F‐test comparing cord length and D_axial_: 1.06, numerator DF: 8, denominator DF: 9, *p*: 0.46, and the F‐test comparing a multivariable model of edema length and cord width: 3.38, DF: 1,8, *p*: 0.10). These results are summarized in Table 3. None of the imaging results predicted D‐waves changes between pre‐injury and 12 weeks post‐injury (Table S1).

**Table 3:** Predictive association of MRI on PTIBS.

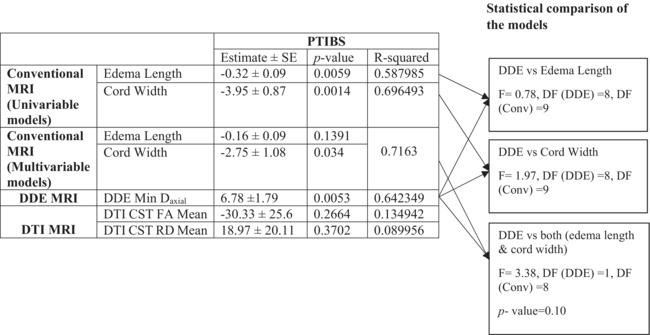

**Figure 8:**
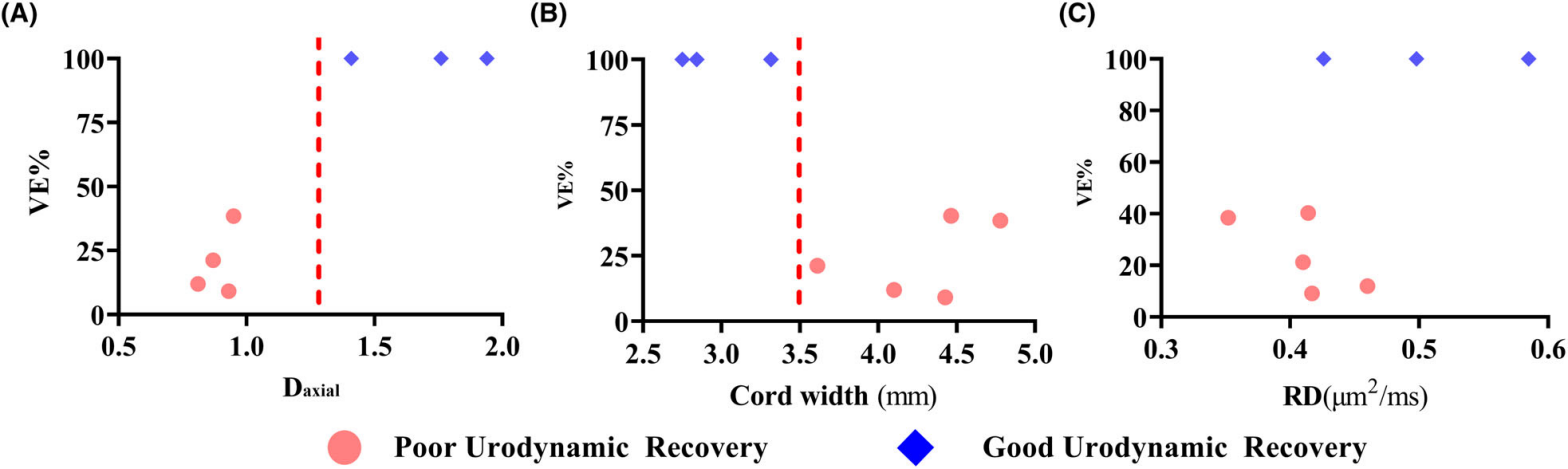
Association of 4 h post‐MRI and bladder voiding efficiency at week‐12 post‐injury.

The analysis of the effect of imaging on voiding efficiency showed that all animals in the poor locomotor recovery cluster grouped together in values of D_axial_ under 1.25 (Fig. 8A), while the good locomotor recovery animals grouped in values over this cut line. On the other hand, the subjects on the poor urodynamic recovery showed cord width values over 3.5 (Fig. 8B), while the good locomotor recovery animals showed results above this value. No obvious cut point was found to differentiate good from poor locomotor recovery animals in regards of DTI (Fig. 8C).

## Discussion

Despite numerous studies, DTI has yet to become a standard of care. In an acute SCI setting, the interpretation of DTI parameters such as fractional anisotropy is confounded by edema, hemorrhage, and inflammation. fDWI was developed to overcome some of these limitations of DTI in acute injury to effectively filter or suppress extracellular signals from vasogenic edema using high diffusion weighting perpendicular to the spinal cord axis. The subsequent detection of diffusion parallel to the cord (D_axial_) with filter is believed to be more reflective of axonal injury associated with focal varicosities or beading that occurs in injured axons.^48^ A handful of published rodent studies have shown fDWI is more accurate than DTI in predicting postinjury locomotor scores.^20^, ^44^ In agreement with these studies, we found that the D_axial_ was significantly diminished 4 h after injury and strongly predicted locomotor and lower urinary tract (LUT) recovery (Fig. 8A). Interestingly, the extent of D_axial_ decreases expanded to all 6 slices at 12 weeks post injury, which suggests ongoing degeneration of axons although the presence of inflammatory cells such as activated microglia may influence D_axial_ as well in long‐term evolving degenerative pathologies. D_radial_, which is obtained from the fDWI protocol but does not include the filtering and is therefore akin to RD derived from DTI, did not change much at short‐term but changed more at long‐term. This suggests that degeneration occurred mostly at the injury site during the first 4 h from the injury, and there was degeneration after 4 h from injury both at the injury site and beyond it. We did not compare fDWI with DTI at the injury site, but we did evaluate the effects of DTI 6 segments rostral to the injury epicenter. There were DTI changes above injury consistent with Wallerian degeneration in all WM tracts at 12‐weeks post‐injury, but not so at 4 h post‐injury. These are in agreement with other studies that report DTI changes at sites remote to injury epicenter.^49^, ^50^

Structural edema has always been considered a good predictor of outcomes after SCI.^51^, ^52^ Not surprisingly, our study confirmed that structural edema in an ultra‐early phase was a strong predictor. fDWI was statistically equivalent, explaining 64% of the variance. These studies showed that fDWI obtained at 4 h post‐injury has significant predictive value and is in contrast to many other human MRI studies that obtained MRIs within such timeframes. These results agree with prior studies in a rodent cervical SCI model,^20^ which reported that edema was the strongest predictor of locomotor outcomes, with fDWI superior to DTI.

The present study demonstrated the detrimental effects of SCI on urinary functions. It was also demonstrated how different grades of injury produce a different level of bladder recovery after 12 weeks (Fig. 7). All these manifestations are compatible with detrusor sphincter dyssynergia (DSD), which is the impaired coordination between detrusor and sphincter during voiding due to a neurologic abnormality.^53^ This syndrome is commonly seen in complete supra‐sacral spinal cord injuries.^54^, ^55^, ^56^ The presence of DSD in these animals, even if they have incomplete SCI, is indicative of the disconnection between the pontine micturition center and the sacral centers for the control of the LUT.^57^, ^58^ The level of spinal cord damage and edema detected by D_axial_ and cord width values obtained on the early stages after SCI correlates with the further urodynamic performance 12 weeks post‐injury. The persistence of neural pathways connecting the cerebral structures and the lumbosacral controlling the LUT can explain the good urodynamic performance in those animals with less damage. However, the underlying inflammation and tissue damage revealed MRI techniques can also play an important role on the plasticity of the spinal pathways controlling the lower urinary tract following SCI.^59^, ^60^ Being able to predict the presence of DSD in these animals with incomplete SCI using MRI biomarkers would be a step forward in the diagnosis and prognosis of urinary manifestations in patients with SCI.

We measured motor‐evoked potentials using D‐waves in the epidural space above and below the injury rather than in the muscle. In contrast to Jutzeler et al.^61^ who also used a 20 cm injury model at T10 but detected no motor evoked potentials (MEPs) in the muscle. Although amplitudes were diminished and latencies prolonged after injuries, there were no differences between good and poor recoveries. Failure to identify could be due to the small sample size. It is also possible that D‐waves may not be sensitive enough to distinguish gradations of incomplete SCI. All 20 cm injuries had D‐waves present below the injury, which contrasts with the Jutzeler study.^61^ The presence of D‐waves is thought to indicate direct corticospinal tract connectivity from the cortex to the site of recording.^37^, ^62^ The neuroanatomical of corticospinal tract in the Yucatan pig remains poorly understood with some studies suggesting terminations at lower cervical or at T1.^62^ Del Cerro showed numerous projections of CST from the primary motor cortex to T6 level in species of pig.^63^ The exact anatomy in Yucatan pig remains unknown although our results suggest the presence of the corticospinal tract to levels lower than T6.

Small sample size is a major limitation of the study. A limited number of long‐term animals were used to evaluate the fDWI information, and in good recovery pigs only 3 animals were considered for urodynamic output at week‐12 post‐injury condition. Moreover, in our study no complete injury was included.

Using high‐resolution inner‐volume diffusion MRI, we have demonstrated the feasibility of high‐quality microstructural imaging in the thoracic spinal cord of Yucatan pig. However, acquiring interpretable DTI data at the injury site is still challenging, hence DTI was only evaluated at sites remote from the injury epicenter. Moreover, we tried to quantify spared WM/GM in either the axial anatomical or fDWI images at the injury epicenter. However, given the limited image resolution, it was not meaningful in our data.

Histopathological findings, though highly valuable to demonstrate neuropathological changes in the WM and GM after injury and could be correlated with MRI and D‐wave findings, were not considered in this study since the goal was to predict later functional recovery from acute MRI.

In acute imaging of a large animal SCI model, fDWI correlated strongly with, though not outperforming, known conventional MRI predictors of locomotor recovery and urodynamic outcomes. Our results serve as an important stepping stone to extend the evidence of fDWI in small animal SCI model to clinical application.

## Author Contributions

A. R. U., K. J. W., D. S., S. L., N. M. J., B. R., U. B., H. C., B. M., X. J., and B. M., designed and conceptualized the study. A. S., G. D., K. C., A. D. M., A. R. U., S, M., N. M. J., and M. M., contributed in data collection. A. R. U., B. U. and A. D. M. statistically analyzed the data. A. R. U., A. D. M., K. J. W., U. B., H. C., B. M., X. J., and B. M., interpreted the data. B. M., acquired funding for the study. All authors critically reviewed and approved the final version of the manuscript.

## Conflict of Interest Statement

The authors declared no conflicts of interest with regards to the current study.

## Supporting information


Supplementary Fig. 1
