# Supplementary material for: Predictive values of spinal cord diffusion magnetic resonance imaging to characterize outcomes after contusion injury
Source: Ann Clin Transl Neurol. 2023 Jul 27;10(9):1647–61. doi: 10.1002/acn3.51855 (PMC10502634; doi:10.1002/acn3.51855)
Supplement: Supplementary file 1 — Supplementary Fig. 1 [file ACN3-10-1647-s001.docx]

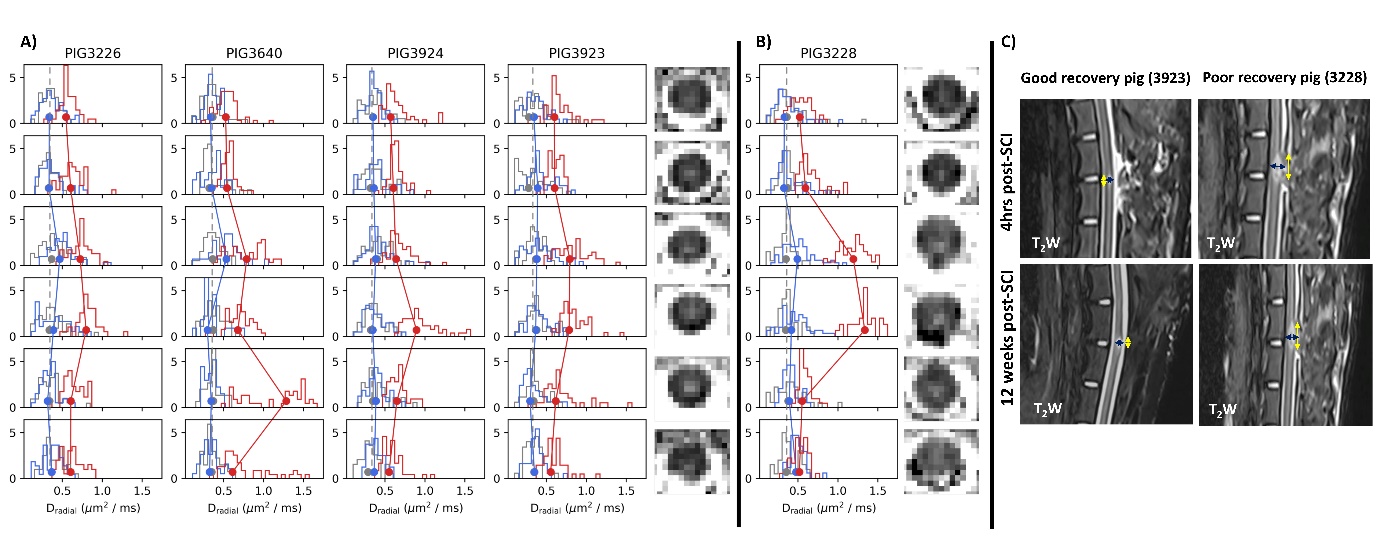


**Supplementary Fig. 1**: fDWI D_radial_ distributions of good recovery cases (left panel) and a poor recovery case (right panel) and representative D_radial_ maps. The rows are slices. The histograms are the distributions of D_radial_ measures in the corresponding slice's whole spinal cord ROI. The y axes are probability density (i.e., the area under each histogram is 1). The gray, blue, and red colors represent preinjury, short-term, and long-term. The dots are median values in the ROI and they were connected by line segments for visualization.


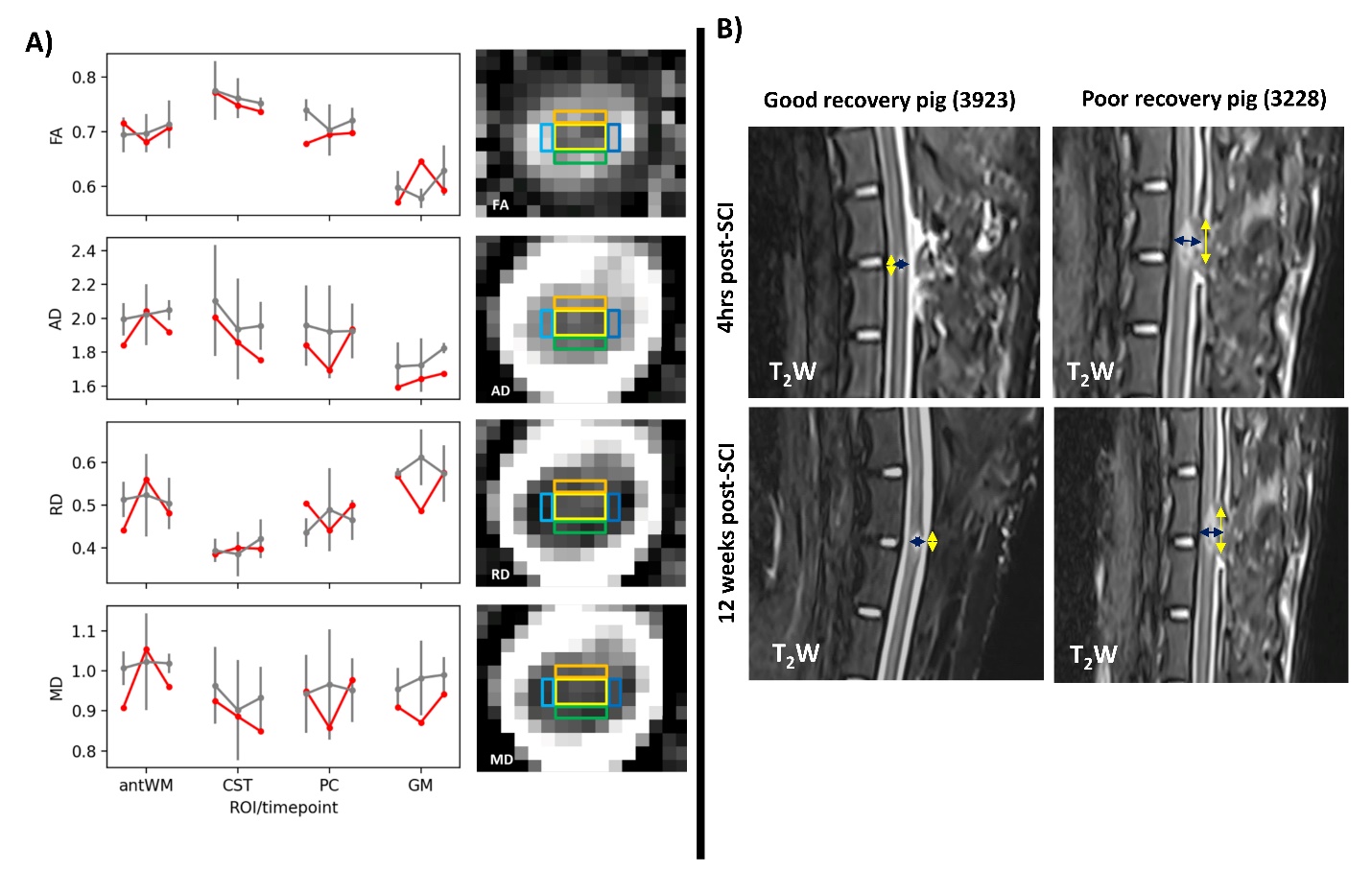


**Supplementary Fig. 2:** **A)** The longitudinal changes in DTI measures (connected gray or red line segments) on anterior WM, CST, PC, and GM (left panel) and representative DTI maps with anterior WM (orange), CST (blue), PC (green), and GM (yellow) ROIs. Gray line segments are means and standard deviations of good recovery cases (n = 4), while red line segments are of the poor recovery case (n = 1). **B)** Conventional MRI lesion of two representative pigs from two different groups at 4 hours post-SCI and 12 weeks post-SCI. The yellow line indicates the edema length and the dark blue line represents the cord width.


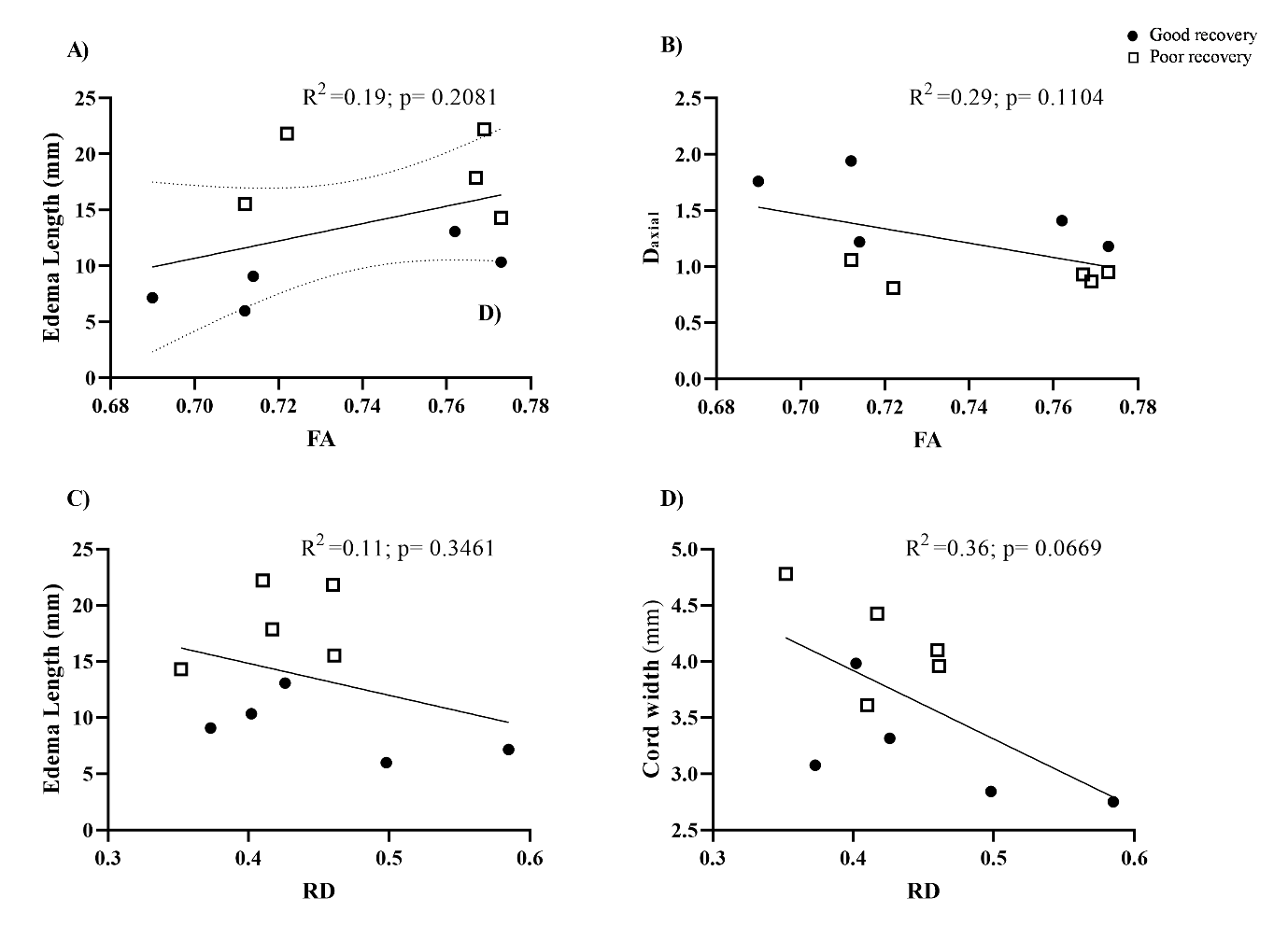


**Supplementary Fig**. **3:** Correlation of conventional MRI, fDWI, and DTI.

**Supplementary Table 1**: Predictive association of MRI on D-waves

|  | | | **Pre-Post Change** | | |
| --- | --- | --- | --- | --- | --- |
|  |  |  | **Estimate ± SE** | **p-value** | **R-squared** |
| **Latency**  **above** | **Conventional MRI** | Edema Length | 0.04 ± 0.05 | 0.4336 | 0.104933 |
|  |  | Cord Width | -0.13 ± 0.62 | 0.8464 | 0.00677 |
|  | **DDE MRI** | D_axial_ | -1.25 ± 1.89 | 0.5374 | 0.080526 |
|  | **DTI MRI** | FA mean | -3.83 ± 9.09 | 0.6884 | 0.028701 |
|  |  | RD mean | 10.72 ± 10.16 | 0.3323 | 0.156331 |
| **Latency**  **below** | **Conventional MRI** | Edema Length | 0.03 ± 0.05 | 0.501 | 0.078684 |
|  |  | Cord Width | -0.12 ± 0.6 | 0.8428 | 0.007096 |
|  | **DDE MRI** | D_axial_ | -0.79 ± 1.85 | 0.6871 | 0.035188 |
|  | **DTI MRI** | FA mean | -3.03 ± 8.82 | 0.7429 | 0.0119285 |
|  |  | RD mean | 7.94 ± 10.18 | 0.4652 | 0.09199 |
| **Amplitude**  **above** | **Conventional MRI** | Edema Length | 1.52 ± 0.94 | 0.157 | 0.303481 |
|  |  | Cord Width | 11.76 ± 13.51 | 0.4174 | 0.112163 |
|  | **DDE MRI** | D_axial_ | -46.28 ± 34.83 | 0.2413 | 0.261039 |
|  | **DTI MRI** | FA mean | 13.88 ± 210.94 | 0.9497 | 0.000721 |
|  |  | RD mean | 79.47 ± 251 | 0.7623 | 0.016434 |
| **Amplitude**  **below** | **Conventional MRI** | Edema Length | 0.49 ± 0.38 | 0.2471 | 0.215052 |
|  |  | Cord Width | -0.58 ± 5.51 | 0.9193 | 0.001858 |
|  | **DDE MRI** | D_axial_ | -7.46 ± 17.44 | 0.6866 | 0.035304 |
|  | **DTI MRI** | FA mean | 33.41 ± 80.01 | 0.6908 | 0.02824 |
|  |  | RD mean | 91.88 ± 89.83 | 0.3458 | 0.14848 |
